# A promising growth promoting *Meyerozyma caribbica* from *Solanum xanthocarpum* alleviated stress in maize plants

**DOI:** 10.1042/BSR20190290

**Published:** 2019-10-25

**Authors:** Farzana Gul Jan, Muhammad Hamayun, Anwar Hussain, Amjad Iqbal, Gul Jan, Sumera Afzal Khan, Hamayoon Khan, In-Jung Lee

**Affiliations:** 1 Department of Botany, Garden Campus, Abdul Wali Khan University Mardan, Mardan, Pakistan; 2 Department of Agriculture, Garden Campus, Abdul Wali Khan University Mardan, Mardan, Pakistan; 3 Centre of Biotechnology and Microbiology, University of Peshawar, Peshawar, Pakistan; 4 Department of Agronomy, Agricultural University Peshawar, Peshawar, Pakistan; 5 School of Applied Biosciences, College of Agriculture and Life Sciences, Kyungpook National University, Daegu, South Korea; 6 Research Institute for Dok-do and Ulleung-do Island, Kyungpook National University, Daegu, Republic of Korea

**Keywords:** abscisic acid, indole-3-acetic acid, M. caribbica, maize, phenols & flavonoids, salt stress

## Abstract

Fungal endophytes are known to secrete a large array of secondary metabolites (phenols, flavonoids, indole acetic acid (IAA) etc.) that facilitate crops under stress conditions. Considering this, a potent plant growth promoting endophyte (SXSp1) from the spines and leaves of *Solanum xanthocarpum* L. has been isolated. The isolated strain ably secreted high quantities of indole-3-acetic acid, phenols and flavonoids. Also, it exhibited phosphate solubilization, siderophore and had 2,2 diphenyl-1-picrylhydrazyl (DPPH) scavenging activity. The SXSp1 also resisted the salinity stress up to 150 mM. LC/MS analysis of SXSp1 culture filtrate (CF) revealed the presence of *p*-hydroxyl benzoic acid, diadzein, genistien, myricetin and caffeoyl-d-glucose. Moreover, the inoculation of maize plants with SXSp1 significantly (*P*=0.05) promoted the chlorophyll and carotenoid contents, root and shoot lengths, plant fresh and dry weights, catalase and peroxidase activities, proline, phenolics, flavonoids and relative water contents (RWCs) under salinity. More interestingly, SXSp1-associated plants showed lower endogenous abscisic acid (ABA) and higher endogenous IAA contents that helped the plants to resist salinity stress up to 100 mM. After sequencing, internal transcribed spacer (ITS) regions (ITS1 and ITS4) and phylogenetic analysis, the SXSp1 was identified as *Meyerozyma caribbica*.

## Introduction

Maize (*Zea mays* L.) is one of the major agricultural crops that can serve as an energy source for humans and animals [1]. U.S.A., Mexico, Nigeria, France and Hungary are the main producers of maize. In Pakistan, maize is grown in various parts of the country to produce a variety of food products, such as tortillas, chips and popcorns. However, a decline in its production has been noticed in the last few years due to the environmental change. Besides its biological importance, maize is very sensitive to both biotic and abiotic stresses, including salinity. Salinity has been reported to affect maize growth and development negatively [2,3]. In fact, the issue of salinity is becoming a global concern as more than 70 countries of the world have been affected. It is estimated that 2000 ha of the agricultural land per day is deteriorating due to salinity [4]. According to the survey reports, salinity has affected ∼6.5 million ha of irrigated land in Pakistan [5]. A continuous change in the climate and high water losses from land by evaporation increase the incidence of salinity in soil, which in turn effects the water uptake by plant roots [6]. Under severe salinity stress plants have disturbed metabolism, accumulate excessive reactive oxygen species (ROS) and stunted growth [7].

To regulate crop yield losses in maize and other cash crops due to salinity, tangible measures are required. Endophytic fungi residing in plant tissues can serve the purpose at their best [8–10]. Certainly, the use of endophytic fungi to alleviate salt stress in an environmental-friendly way seems to be one of the best solutions to the problem. Endophytes can secrete plant growth regulating hormones (gibberellins, auxin, cytokinin, abscisic acid (ABA)) and secondary metabolites (phenols and flavonoids) to help plant against salinity stress [11–18]. Indole acetic acid (IAA) is known to control root growth, provoke ethylene-based abscission, hinder H_2_O_2_ production and encourage general plant growth under salinity stress [19]. The flavonoids control stomatal opening and resource allocation during stress conditions [20]. Various studies in past have confirmed that IAA, 2,2 diphenyl-1-picrylhydrazyl (DPPH) activity, phenols and flavonoids are the key determinants by which endophytes help the host plants to thrive in salt affected soil [21,22]. Though, researchers have demonstrated the positive effect of endophytes on plant growth under stress conditions, yet the field is still open to discover novel endophytic strains. In this context, we have explored the role of endophytic *Meyerozyma caribbica* in mitigating salt stress in maize seedlings.

## Methods

### Isolation of endophytic fungi

In the present study, eight endophytic fungal strains (SXSp1, SXSp2, SXSp3, SXSp4, SXSp5, SXSp6, SXSp7 and SXSp8) were isolated from the spines and leaves of *S. xanthocarpum* collected from the district Mardan. The district lies between 34°05′ to 34°32′ north latitudes and 71°48′ to 72°25′ east longitudes. The average temperature of Mardan is 23.2°C and average rainfall is 110 mm. The texture of the soil ranges from sandy loam to clayey, having a pH from 5.9 to 8.1.

Initially, spines and leaves were washed with tap water and surface sterilized with 70% ethanol for 1 min in laminar flow hood. The treated spines and leaves were then washed with double distilled water (ddH_2_O) to remove traces of ethanol. To isolate endophytic fungi, the spines and leaves were cut into 0.5-cm segments and were placed on Hagem media [23] supplemented with streptomycin (inhibiting bacterial growth). The plates were incubated for 3 days at 28°C. A parallel experiment was also carried out with intact leaves and spines to confirm the surface sterilization [24]. Colonization frequency of each fungal isolate was calculated [25]. The fungal isolates collected from the Hagem media were subcultured on PDA and checked for purity. The subculturing was repeated till the establishment of axenic cultures.

### Colonization frequency

The colonization frequency of isolated endophytic fungi from the spines and leaves was calculated according to the protocol of Gond et al. [26].


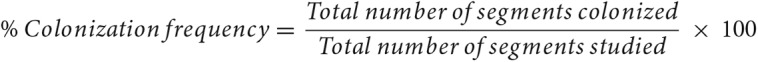


### Salt stress tolerance

Fungal isolates were taken from PDA master plates and inoculated in 50 ml Czapek-broth (Peptone 10 g/l, Glucose 10 g/l, 0.5 g/l MgSO_4_.7H_2_SO_4_, 0.5 g/l KCl and 0.01 g/l FeSO_4_.7H_2_SO_4_ pH 7.3 ± 0.2) supplemented with 100, 150 and 200 mM NaCl. The cultures in the flasks were then incubated for 7 days at 28°C and at 120 rpm [27]. After 7 days of incubation, fresh and dry biomass (g) of the inoculated fungal isolates were checked [28].

### Determination of IAA through GC/MS

Fungal culture filtrate (CF) was subjected to a gas chromatography-mass spectroscopy (GC/MS-SIM) [29]. The CF of the isolate was taken in a test tube and filtered through 0.45-μm cellulose acetate filter. The pH of filtrate was adjusted to 3.0 with 1 N HCl and then extracted with ethyl acetate. The organic layer was evaporated at 45°C in a water bath. The dried sample was then dissolved in 5 ml of 0.1 M acetic acid and eluted through the reverse phase C18 column, using 30, 50 and 100% methanol. Methanol from the eluted samples was evaporated at 45°C using a water bath. The residues left were dissolved in 1 ml methanol and added 1.5 ml ethereal diazomethane to prepare methyl ester fraction. The methylated samples were re-dissolved in ethyl acetate before being analyzed by GC/MS-SIM (6890N network GC system, and 5973 network mass selective detector; Agilent, Palo Alto, CA, U.S.A.). For IAA, 1 μl sample was injected in a DB-1 capillary column (J&W Scientific Co., Folsom, CA, U.S.A.). The GC oven temperature was programmed as: holding temperature of 70°C for 2 min, then rose to 200°C (with a steady increase in temperature, i.e. 20°C/min) and finally reached 285°C (with an increase of 5°C/min). Helium as a carrier gas was maintained at a head pressure of 30 kPa. The GC was directly interfaced to a mass selective detector with an interface and source temperature of 230°C, an ionizing voltage of 70 eV and a dwell time of 100 min.

### Phosphate solubilization

Solubilization of phosphate was detected on Pikovskaya’s agar plates [30]. Initially, the isolated fungal strains were cultured on Pikovskaya agar media (10 g/l dextrose, 0.5 g/l yeast extract, 0.5 g/l ammonium sulfate, 5 g/l calcium phosphate, 0.2 g/l potassium chloride, 0.0001 g/l manganese sulfate, 0.1 g/l magnesium sulfate, 0.0001 g/l ferrous sulfate and 15 g/l agar) containing tri-calcium phosphate as insoluble phosphate source and incubated at 27°C for 48 h to observe a clear zone around the tested strains [30].

### Siderophores activity

The siderophores activity was determined by the inoculation of endophytes on Chrome Azurol S (CAS) plates by the method of Schwyn and Neilands [31]. The appearance of orange halos by the isolated strain indicates siderophores production.

### Total phenolics determination in the CFs of isolated fungal strains

The total phenolics were determined by following the method of Qawasmeh et al. [32]. Total polyphenols were determined by the Folin–Ciocalteau method. Plant tissues (100 mg) were ground with 80% ethanol and the resultant extracts (0.5 ml) were mixed with Folin–Ciocalteau reagent (0.5 ml) and 10% Na_2_CO_3_ (0.5 ml). The absorbance of the reaction mixture was measured at 760 nm after 1 h of incubation at room temperature. A blue coloration indicates the presence of phenol and their absorbance was checked at 650 nm using SHIMADZU spectrophotometer (Kyoto, Japan). Gallic acid was used as a standard.

### Total flavonoids determination in CFs of isolated fungal strains

Colorimetric determination of flavonoids was carried out by following the protocol of Srinivasan et al. [33]. Sample (1 ml) was mixed with 4 ml of distilled water and 0.3 ml of sodium nitrite solution (5% w/v); 0.3 ml of aluminium chloride solution (10%) was then added to the mixture, followed by the addition of 0.2 ml of NaOH (1 M) after 1 min. The volume was made up to 10 ml with distilled water and the contents were mixed thoroughly. A white milky coloration indicated the presence of flavonoids. Quercitin standard curve was used for flavonoids determination.

### Screening of fungal isolates in relation to plant growth promotion

The growth promoting ability of salt-tolerant fungal strains was assessed on Waito-c rice in pots. Seeds of Waito-c rice were sterilized with (70%) ethanol and washed thrice with autoclaved distilled water. Petriplates having two layers of autoclaved filter papers were used to keep the seeds of Waito-c rice for 3 days in an incubator operated at 27°C. After 3 days, the uniform germinated seedlings were transferred to pots containing 0.8% agar water media. Once the Waito-c rice seedlings established in the pots, 10 ml of fungal spore suspension (10^6^ cfu/ml) was applied at the apex of each seedling. The seedlings were then allowed to grow for the next 2 weeks at 27°C to develop fungi–plant relationship. After 2 weeks, the growth was checked and based on the efficiency of the host plant growth promotion, the most efficient fungal strain was selected for further study.

### DPPH% activity in the CF of selected SXSp1

The scavenging activity for DPPH free radicals was measured in fungal CF with or without salinity stress [34]. De-colorization of DPPH was determined by measuring the decrease in absorbance at 517 nm against methanol. Methanol was used as a blank, whereas ascorbic acid was used as a positive control.

### LC MS/MS analysis of compounds in the CF of selected SXSp1

The fungal culture having the ability to exhibit antioxidant activity, were further evaluated by LC MS/MS (LTQ XL, Thermo Electron Corporation, U.S.A.) for the presence of bioactive compounds [35]. The detection was performed through direct injection mode with Electron Spray Ionization (ESI) probe, at a positive-mode. The capillary temperature was kept at 280°C, while the sample flow rate was set at 8 μl/min. The mass range was selected from 50 to 1000 m/z. The collision-induced dissociation energy (CID) during MS/MS was kept in the range of 10–45, depending upon the nature of the parent molecular ion. As a mobile phase, the ratio of methanol and acetonitrile was 80:20 (v/v) for the HPLC fractionation. The MS parameters for each compound were optimized to ensure the most favorable ionization, ion transfer conditions. The optimum signal of both the precursor and fragment ions was attained by infusing the analytes and manually tuning the parameters. The source parameters were identical for all of the analytes.

### Isolation of fungal DNA

DNA isolation and PCR of selected SXSp1 strain was performed, according to an established protocol of Khan et al. [36]. Fungal mycelium (200 mg) were collected from fresh culture, suspended in 500 μl of a bead beating solution with the addition of 5% sodium dodecyl sulfate in micro-centrifuge tubes. The tubes were then vortexed and centrifuged for 10 min at 11000×***g*** at 4°C. The supernatants were decanted into new tubes and equal volume of phenol:chloroform:isoamyl alcohol (25:24:1) were added to each sample, again vortexed and centrifuged for 5 min. The aqueous layer was transferred to a new tube and an equal volume of chloroform:isoamyl alcohol (24:1) were added to it. The tubes were then centrifuged for 5 min at 10000×***g***. The supernatant was transferred to new Eppendorf tubes, and 2.5 volumes of isopropanol were added for the precipitation of DNA. The tubes were incubated in a refrigerator for 1 h, and centrifuged for 10 min at 14000×***g***. The pellets were washed twice with cold 70% ethanol, air-dried and added 40 μl TE buffer. The purity of the extracted DNA and its quantity was measured by Thermo Scientific NanoDrop spectrophotometer at 260 nm [37].

### Identification of endophyte by internal transcribed spacer sequence homology

Selected endophytic fungal strain was identified by amplifying internal transcribed spacer (ITS) region of 18S rDNA with universal primers, ITS-1; 5′-TCC GTA GGT GAA CCT GCG G-3′ and ITS-4; 5′-TCC TCC GCT TAT TGA TAT GC-3′ [38]. The PCR was performed with 20 ng of genomic DNA as a template in a 30-μl reaction mixture by using an EF-Taq (SolGent, Korea). The PCR was programmed as follows: 95°C for 2 min; 35 cycles (95°C for 1 min, 55°C for 1 min and 72°C for 1 min); 72°C for 10 min. The PCR products along with DNA markers (DNA ladder) were then loaded on to an agarose gel and subjected to electrophoresis for 30 min. The gel was finally stained with 0.01 g/ml Ethidium Bromide and examined under UV transilluminator lamp.

Approximately, 1600 bp purified PCR products were sequenced with 18S rDNA region by using universal primers, ITS-1; 5′-TCC GTA GGT GAA CCT GCG G-3′ and ITS-4; 5′-TCC TCC GCT TAT TGA TAT GC-3′ accomplished through a Big Dye terminator cycle sequencing kit v.3.1. Both PCR sequencing and amplification was analyzed by an automated DNA sequencing system (Applied Biosystems, Foster City, U.S.A.) at the Macrogen, Inc., Seoul, Korea.

The sequence obtained was subjected to Blastn1 for sequence homology estimation. The selected sequences obtained as a result of homology search was then inferred by using the Max-parsimony method based on Tamura and Nei [39] model. The analysis involved 16 nucleotide sequences and the evolutionary analysis were conducted in MEGA7 [40].

### Plant growth promotion and salt stress alleviation potential of the selected SXSp1

Healthy and uniform seeds (30 seeds) of *Z. mays* were surface sterilized by dipping them in 0.1% HgCl_2_ followed by washing with ddH_2_O. Seeds were then allowed to germinate in Petriplates on wet filter papers in order to get equally germinated seedlings. After germination, seedlings were transferred to pots (pot width = 9 cm and length = 11.3 cm) containing autoclaved soil and allowed to establish before the fungal biomass was added to the soil. Soil inoculation was performed by drench inoculation method which involved the application of fresh biomass of selected fungal strain SXSp1 (0.3 g/300 g soil) around the root zone in order to assess their ability of alleviating salinity stress in maize [41]. Salinity stress of 25 ml water containing 100 mM NaCl, KCl and K_2_SO_4_ was applied after every 3 days for next 20 days. The pots had no pores to allow leakage of water and salts. The plants were watered daily. The experiment was terminated after completion of salt stress and plant growth attributes were analyzed [42]. The plant dry biomass was measured after drying the plants at 70°C for 48 h in an oven [43]. Chlorophyll a (Chl a), chlorophyll b (Chl b) and carotenoids was determined in the extract of fully expanded maize leaves by MacKinney equations [44]. Fully expanded fresh leaves of fungal inoculated and non-inoculated maize plants under salt stress were homogenized with 2 ml of acetone (80%) and washed twice to reach a final volume of 7 ml. The absorbance was measured using a spectrophotometer at 480, 645 and 663 nm. Electrolyte leakage (EL) was determined by adopting standard method [45]. Leaf relative water contents (RWCs, %) were measured by following the standard protocol [46]. For the determination of peroxidase activity (POD), Catalase, proline, IAA and ABA, maize seedlings were cut into pieces and frozen immediately in liquid nitrogen and stored in freezer at −70°C.

### Quantification of endogenous IAA and ABA in maize

Extraction and purification of plant hormones were analyzed by using HPLC according to the method of Kettner and Dörffling [47]. Fresh leaves (1 g) were ground at 4°C in 80% methanol with butylated hydroxy toluene (BHT) as an antioxidant (2 mg/100 ml) [48]. After 72 h extraction, the pooled extract was centrifuged at 3000 rpm and the supernatant was partitioned at pH 2.5–3 with ethyl acetate (1/4th volume of the extract). The ethyl acetate phase was dried down completely on the rotary thin film evaporator [18] and the residues were re-dissolved in 100% methanol. The samples were then passed through a Millipore filter (0.45 μ) and were analyzed by HPLC (Agilent 1100), equipped with variable UV detector and C18 column (39 × 300 mm) (BondaPack Porasil C18, 37/50 μm, Waters, Eschborn, BRD). Methanol and water in the ratio of (30:70; v/v) were used as mobile phase at a flow rate of 1500 μl/min and a run time of 20 min/sample. Pure IAA and ABA was used as standards.

### Estimation of total proline in maize

The proline contents of maize seedling was measured according to the method described by Shaw et al. [49]. The fresh leaf samples (0.3 g) were homogenized in 4 ml (3%) of aqueous sulfosalicylic acid and the homogenate was subjected to centrifugation at 12000 rpm for 10 min. Equal volumes of glacial acetic acid and ninhydrin were added to the supernatant. The mixture was boiled on a water bath adjusted at 100°C for 1 h and then extraction was done with 4 ml of toluene. The absorbance was measured at 520 nm using toluene as a blank.

### Determination of catalase activity in maize

Leaves of maize seedlings (20 mg) were collected and homogenized in 50 mM Tris/HCl buffer (pH 7.0) containing 1 mM EDTA, 3 mM MgCl_2_ and 1.0% PVP. The homogenate was centrifuged at 15000 rpm and 22°C for 15 min. The resultant supernatant was used in enzymatic assays. Catalase activity was assayed by previously described method [50]. To the crude supernatant, a mixture (0.5 ml) of 0.2 M H_2_O_2_ and 10 mM phosphate buffer (pH 7.0) was added. A decrease in the absorbance at 240 mm was noted.

### Estimation of POD in maize

POD was assayed by following the established protocol [51]. Fresh leaves (200 mg) were subjected to homogenization in 0.1 M phosphate buffer (pH 6.8), followed by centrifugation at 17000 rpm and 2°C for 15 min. The collected supernatant (0.1 ml) was then mixed with 0.1 M phosphate buffer (pH 6.8), 50 μl H_2_O_2_ and 50 μl pyrogallol. The reaction mixture was incubated at 25°C for 5 min and the reaction was stopped by adding 0.5 ml of 5% (v/v) H_2_SO_4_. The absorbance was measured at 420 nm.

### Estimation of total phenolics and flavonoids in maize

Cai et al. [52] procedure was applied for the analysis of phenolics in maize. Different concentrations (100, 200, 300, 500 and 600, 700 and 900 mg/ml) of Gallic acid (Sigma–Aldrich) were used to make a standard curve. El-Far et al. [53] method was applied for the analysis of total flavonoids in maize. A standard curve was made by using different concentrations of quercetin (15, 30, 60, 120, 240 and 480 μg/ml, Sigma–Aldrich) and the OD was measured at 415 nm.

### Statistical analysis

Data analyses were done using SPSS 20 for Windows. Mean values among treatments were compared by Tukey’s honestly significant difference (HSD) test at *P*=0.05.

## Results

### Isolation of fungal endophytes from *S. xanthocarpum*

After the inoculation of *S. xanthocarpum* spines and leaves, a total of eight fungal endophytes had been isolated and identified. The results revealed that the colonization of the endophytes on spine samples had been high as compared with the leaf samples. Out of the eight isolated strains, SXSp1 showed plant growth promotion, whereas SXSp5, SXSp6 and SXSp7 inhibited the plant growth and SXSp2, SXSp3, SXSp4 and SXSp8 had no effect (Table 1).

**Table 1:** Screening of the isolated endophytic fungal strains from the spines and leaves of *S. xanthocarpum*

Strains	Colonization (%)		Growth under salt stress (NaCl)			Growth promotion
	Spines	Leaves	100 mM	150 mM	200 mM	
SXSp1	100	25	+	+	-	+
SXSp2	68	22	+	-	-	N
SXSp3	60	15	+	-	-	N
SXSp4	14	14	-	-	-	N
SXSp5	82	55	-	-	-	-
SXSp6	13	-	-	-	-	-
SXSp7	20	-	-	-	-	-
SXSp8	40	11	+	-	-	N
SXSp1	100	25	+	+	-	+

### Salinity stress resistance of the fungal isolates

The response of fungal isolates to various salt concentrations has been studied in the form of visible fungal growth in Czapek broth supplemented with various concentrations (i.e. 100, 150 and 200 mM) of NaCl after 7 days of incubation (Table 1). The strain SXSp1 has been found to be the most resistant strain that grew well in the medium supplemented with 150 mM NaCl. On the other hand, SXSp4, SXSp5, SXSp6 and SXSp7 have failed to resist the salinity stress, while SXSp2, SXSp3 and SXSp8 survived 100 mM of NaCl stress (Table 1).

### Selection of the best strain(s) isolated from *S. xanthocarpum* for further study

The isolated fungal endophytes have initially assessed for IAA, phenolics, flavonoids, P-solubilization and siderophore activities in order to select the best strain(s) for further study. Among the isolated strains, SXSp1 performed well regarding phosphate solubilization, siderophore, IAA, phenolics and flavonoids production (Table 2). The CF of SXSp1 grown in Czapek broth had the highest amount of IAA, i.e. 30 ± 1.7 μg.ml^−1^. The isolated fungal endophytes, SXSp1 ably produced greater quantities of phenols and flavonoids. SXSp1 has also produced siderophore and efficiently mobilized insoluble phosphate present in Pikovskaya’s medium. Likewise, the other tested strains have produced IAA, phenolics and flavonoids, but in significantly lower quantities. Moreover, all the tested strains have shown siderophore activity, except SXSp3 and SXSp7. Moreover, bioassay on Waito-c rice showed that ten strains were plant growth promoter and two strains were growth inhibitors (data not shown). On the basis of IAA, phenolics, falvonoids, P-solubilization, siderophore activities and excellent performance in relation to plant growth promotion of Waito-c rice, the fungal strain SXSp1 was selected for further study.

**Table 2:** Potential of endophytes from *S. xanthocarpum* to exhibit siderophore, P-solubilization, IAA, phenols and flavonoids production

Fungal isolates	IAA (μg/ml)	Phenolics (μg/ml)	Flavonoids (μg/ml)	P-solubilization (μg/ml)	Siderophore
SXSp1	30 ± 1.7^6^	60 ± 1.8^5^	50 ± 1.5^4^	30 ± 1.7^6^	+
SXSp2	11 ± 0.6^3^	12 ± 0.5^1^	14 ± 0.6^3^	11 ± 0.6^3^	+
SXSp3	8 ± 0.5^2^	14 ± 0.7^1,2^	10 ± 0.4^1^	8 ± 0.5^2^	-
SXSp4	13 ± 0.7^3,4^	11 ± 0.5^1^	10 ± 0.4^1^	13 ± 0.7^3,4^	+
SXSp5	20 ± 1.2^5^	16 ± 0.5^2^	12 ± 0.5^2^	20 ± 1.2^5^	+
SXSp6	5 ± 0.3^1^	19 ± 0.8^3^	10 ± 0.4^1^	5 ± 0.3^1^	+
SXSp7	3 ± 0.2^1^	22 ± 1.1^4^	12 ± 0.5^2^	3 ± 0.2^1^	-
SXSp8	14 ± 0.8^4^	23 ± 1.1^4^	09 ± 0.6^1^	14 ± 0.8^4^	+

Each value represents the mean ± SE of three replicates. Means followed by different numbers (superscript (1–6)) are significantly different (Duncan multiple range test; *P*<0.05).

### Aptitude of the SXSp1 in NaCl stress

The ability of the isolated SXSp1 to resist various concentrations of NaCl has been tested using Czapek media. SXSp1 has successfully grown in Czapek media supplemented with NaCl. However, a retarded growth of SXSp1 in terms of fresh and dry weights has been noticed with the increased concentration of salts in the medium (Table 3). Similarly, SXSp1 has significantly lost its potential to produce IAA, phenolics and flavonoids in appreciable quantities. The selected endophytic strain (SXSp1) on the other hand flourished in the presence of 100 mM of NaCl without any significant changes in their fresh and dry biomasses, IAA, phenolics, and flavonoids production (Table 3).

**Table 3:** Effect of salt on SXSp1 biomass, IAA, phenols and flavonoids production

Treatments	FFB (g)	FDB (g)	IAA (μg/ml)	Phenols (μg/ml)	Flavonoids (μg/ml)
Control	4 ± 0.2^2^	1.9 ± 0.2^3^	30 ± 1.7^3^	60 ± 1.8^3^	50 ± 1.5^2^
100 mM	3.7 ± 0.2^2^	1.8 ± 0.1^3^	27.8 ± 1.3^3^	58 ± 3.2^3^	47 ± 2.5^2^
150 mM	1.1 ± 0.1^1^	0.6 ± 0.1^2^	8 ± 0.5^2^	20 ± 1.1^2^	11 ± 0.6^1^
200 mM	1 ± 0.1^1^	0.1 ± 0.1^1^	2 ± 0.1^1^	9 ± 0.5^1^	7 ± 0.4^1^

Each value represents the mean ± SE of three replicates. Means followed by different numbers (superscript (1–3)) are significantly different (Duncan multiple range test; *P*<0.05).

### Analysis of secondary metabolites by LC MS/MS

The CF of SXSp1 that exhibited significantly high DPPH scavenging activity, phenolics and flavonoids contents under salinity stress have been analyzed using LC MS/MS to identify the bioactive metabolites. Diadzein, *p*-hydroxyl benzoic acid, genistien quercetagetin and caffeoyl-d-glucose has been identified as the major phenols and flavonoids in the CF of SXSp1 (Supplementary Figure S1). The chemical characteristics and structures of the identified secondary metabolites are summarized in Table 4.

**Table 4:** Chemical properties of identified phenols and flavonoids in the SXSp1 extract by LC MS/MS

Compound	Molecular formula	Average mass (g/mol)	Chemical structure
*p*-hydroxybenzoic acid	C_7_H_6_O_3_	138.12	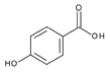
Genistien	C_15_H_10_O_5_	270.241	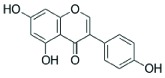
Diadzein	C_15_H_10_O_4_	254.23	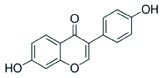
Quercetagetin	C_15_H_10_O_8_	318.23	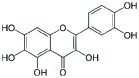
Caffeoyl-d-glucose	C_15_H_18_O_9_	342.3	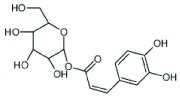

### Molecular identification and phylogenetic analysis

**Figure 1:**
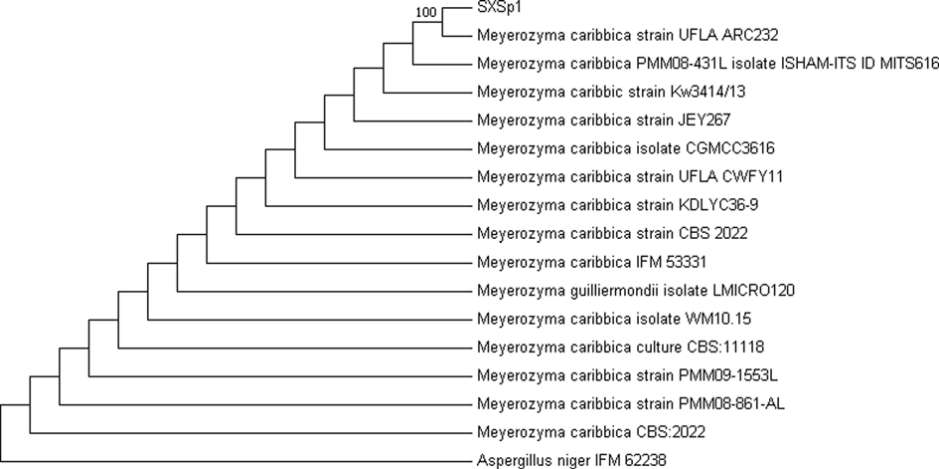
Identification of endophytic fungal isolate SXSp1 by phylogenetic analysis The evolutionary history was inferred by using neighbor joining tree method based on the Tamura and Nei model [39]. The analysis involved 17 nucleotide sequences. Evolutionary analyses were conducted in MEGA7. Bootstrap support of 57 for isolate SXSp1 with *M. caribbica* (99% sequence homology) strongly recommends our fungal isolate as a new strain of *M. caribbica*.

The fungal endophyte SXSp1 has been identified through sequencing of ITS region of 18S rDNA. The fungal isolate has been identified by relating ITS region (including partial sequence of 18S rDNA, complete sequence of ITS1 and ITS2, complete sequence of 5.8S rDNA and partial sequence of 28S rDNA) of the SXSp1 with the associated sequences existing in the GenBank database of NCBI (http://www.ncbi.nlm.nih.gov/BLAST/). The closely related sequences have been recovered from GenBank and subjected to phylogenetic analysis by using MEGA7 to construct the Maximum Parsimony tree. The isolate has been identified as *Meyerozyma caribbica* having 100 bootstrap supports (Figure 1). The *M. caribbica* gene sequence has been submitted to NCBI GenBank under the accession no. MG976725.

### Physicochemical changes in *M. caribbica*-associated maize plants under salinity stress

#### Effect on chlorophyll and carotenoid contents

**Figure 2:**
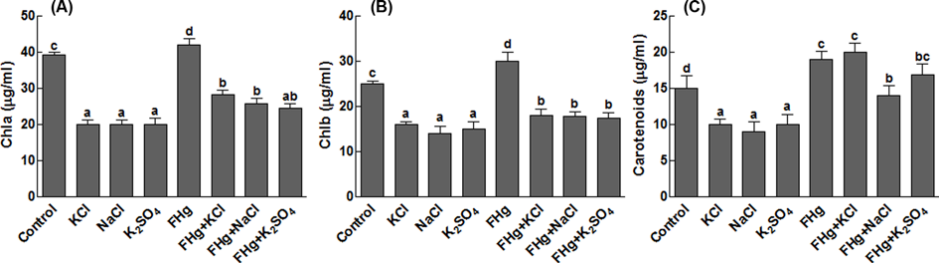
Effect of different salts on chlorophyll and carotenoids contents of maize plants (**A**) Represents chlorophyll a contents; (**B**) represents chlorophyll b contents; (**C**) represents carotenoids’ contents. FHg, fungal endophyte SXSp1; KCl, potassium chloride; NaCl, sodium chloride; K_2_SO_4_, potassium sulfate. Each value represents the mean ± SE of three replicates. Means followed by different letters (a–d) are significantly different (Duncan multiple range test; *P*<0.05).

Salinity stress reduced the chlorophyll and carotenoid contents of the maize plants significantly. However, inoculation of maize plants with *M. caribbica* mitigated the salinity stress (Figure 2). The average increase in 26.2 μg/ml in chlorophyll a (Figure 2A), average increase in 17.76 μg/ml in chlorophyll b (Figure 2B) and an average increase in 16.96 μg/ml in carotenoid contents (Figure 2C) have been noticed in plants inoculated with *M. caribbica* as compared with the non-inoculated maize plants under the salinity stress.

### Effect of *M. caribbica* on growth parameters, EL and RWCs

**Figure 3:**
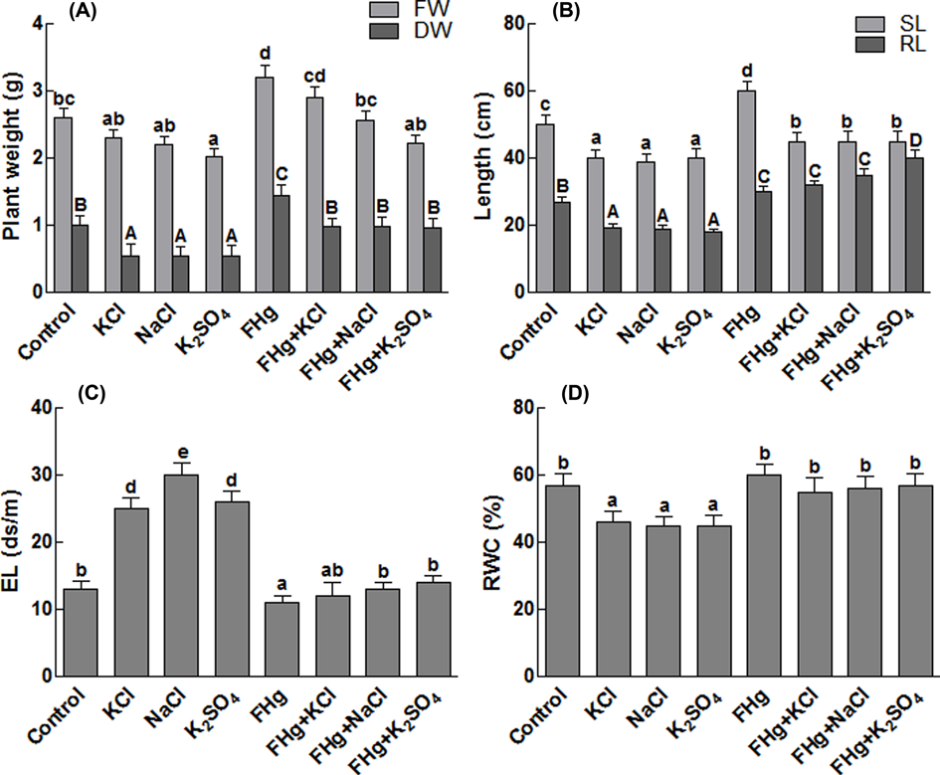
Effect of different salts on on physiochemical characteristics of maize plants (**A**) Represents maize plant weights on dry and wet basis; (**B**) represents root and shoot lengths of maize plants under salt stress; (**C**) represents electrolytic leakage of maize plants under salt stress; (**D**) represents RWC of maize plants under salt stress. DW, dry weight; EL, electrical conductivity; FHg, fungal endophyte SXSp1; FW, fresh weight; KCl, potassium chloride; K_2_SO_4_, potassium sulfate; NaCl, sodium chloride; RL, root length; SL, shoot length. Each value represents the mean ± SE of three replicates. Means followed by different letters (a–e, A–C) are significantly different (Duncan multiple range test; *P*<0.05).

From the results, it is quite evident that the *M. caribbica* inoculated maize plants achieved higher weights on the fresh and dry basis as compared with the non-inoculated controls (Figure 3A). Increase in fresh and dry weights of *M. caribbica* associated maize seedlings without stress was 3.2 and 1.4 g, respectively. Additionally, *M. caribbica* inoculated maize plants under salinity stress (NaCl, KCl and K_2_SO_4_) have significantly higher fresh and dry weights in comparison with the non-inoculated stressed plants. Moreover, the fungal endophytes have enabled the maize plant to stand against the salt stress, which is quite evident from the higher biomass of maize plants inoculated with *M. caribbica* relative to the unstressed control plants (Figure 3A). Figure 3B shows that *M. caribbica* helped the maize plants to gain significantly higher shoot and root lengths under normal conditions as compared with the control. However, salinity stress has significantly decreased the shoot and root lengths of the maize plants by 20 and 30%. However, shoot and root lengths of the maize plants associated with the *M. caribbica* were restored under salinity stress (Figure 3B). The data regarding the EL from maize leaf grown under increased salinity revealed higher EL as compared with the non-saline-control (Figure 3C). A significant reduction (51.61%) in EL of the maize plants under salt stress has been noticed in *M. caribbica* associated plants. Similarly, a significant decrease in EL (15.38%) has been observed, when non-saline control was inoculated with *M. caribbica* (Figure 3C). Besides, un-associated maize plants that have gone through salinity stress had lower RWC compared with the other maize plants (Figure 3D). Conversely, it was quite interesting to observe that the application of endophytes to salt stressed maize seedlings have gained higher RWC. The increase in RWC was 24% higher in maize plants inoculated with *M. caribbica* and exposed to salt stress (Figure 3D).

### Effect of *M. caribbica* on maize endogenous IAA and ABA contents

**Figure 4:**
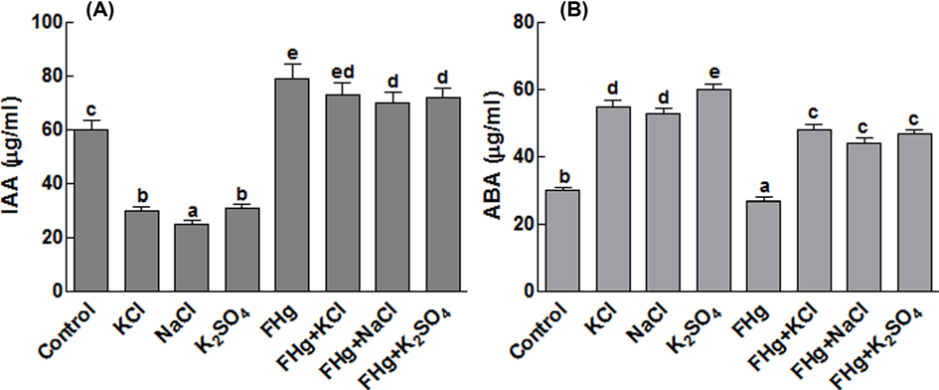
Quantitative changes in IAA and ABA contents of maize under salt stress (**A**) Represents IAA contents of maize plant under salt stress; (**B**) represents ABA contents of maize plants under salt stress. FHg, fungal endophyte SXSp1; KCl, potassium chloride; K_2_SO_4_, potassium sulfate; NaCl, sodium chloride. Each value represents the mean ± SE of three replicates. Means followed by different letters (a–e) are significantly different (Duncan multiple range test; *P*<0.05).

The concentration of endogenous IAA has been determined in endophytes associated and non-associated maize seedlings grown under control (no salt) and saline conditions. The results revealed that the IAA contents of maize plants have increased by 31.67%, when the plants were inoculated with *M. caribbica* compared with the control (Figure 4A). The ABA contents of the maize plants under salt stress have also increased (Figure 4B). However, the application of *M. caribbica* to salinity stressed plants decreased the ABA by approximately 17.2%. On general basis, the *M. caribbica* inoculated plants have significantly lower concentrations of ABA as compared with the control plants under normal conditions (Figure 4B).

### Effect on oxidative stress

**Figure 5:**
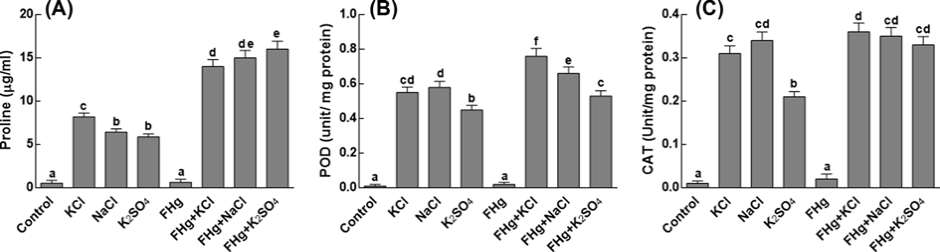
Effect of different salts sources on oxidizing capacity of maize plants (**A**) Represents proline contents of maize plants under salt stress; (**B**) represents peroxide activity of maize plants under salt stress; (**C**) represents catalase activity of maize plants under salt stress. FHg, fungal endophyte SXSp1; KCl, potassium chloride; K_2_SO_4_, potassium sulfate; NaCl, sodium chloride. Each value represents the mean ± SE of three replicates. Means followed by different letters are significantly different (Duncan multiple range test; *P*<0.05).

Proline is an osmoprotectant that has been shown to accumulate in plants in response to salinity stress. In concurrence with this, our results showed that maize colonized with *M. caribbica* produced significantly higher levels of proline under salt stress (Figure 5A). Percent increase in proline content has been two-fold, when salinity-treated maize plants were inoculated with *M. caribbica*. Salinity stress has also caused a significant increase in the activities of antioxidant enzymes, i.e. peroxidases and catalases. Percent increase in POD activity has almost 23%, when salinity stressed maize plants have been inoculated with *M. caribbica* (Figure 5B). Likewise, 25% increase in catalase activity has been recorded in *M. caribbica* associated maize plants as compared with the non-associated maize plants under salt stress (Figure 5C).

### Effect on total phenolics and flavonoids in maize

**Figure 6:**
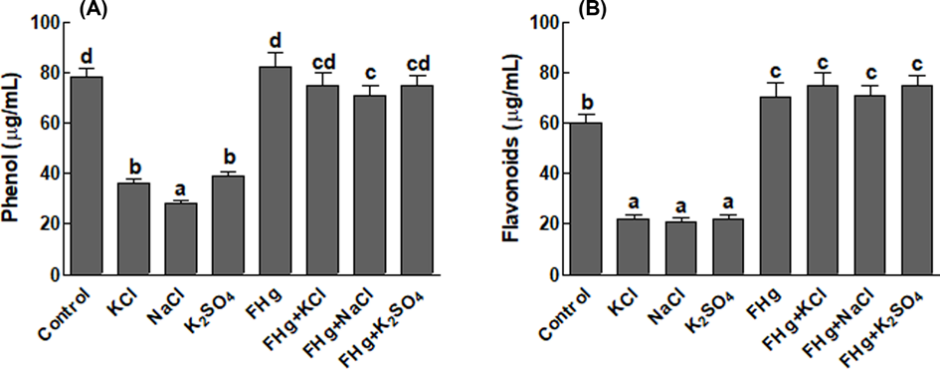
Effect of different salt sources on phenols and flavonoids contents of maize plants (**A**) Represents phenolic contents of maize plants under salt stress; (**B**) represents flavonoids contents of maize plants under salt stress. FHg, fungal endophyte SXSp1; KCl, potassium chloride; K_2_SO_4_, potassium sulfate; NaCl, sodium chloride. Each value represents the mean ± SE of three replicates. Means followed by different letters (a–d) are significantly different (Duncan multiple range test; *P*<0.05).

Total flavonoids and phenolics were decreased in maize plants when subjected to different salt stresses. However, an increase in both phenolics and flavonoids was observed in maize plants inoculated with *M. caribbica* and exposed to salt stress (Figure 6A,B).

## Discussion

The accumulation of salts in crop plants is threatening to human well-being and at the same time, limits crop yield and productivity [54,55]. Currently, endophytes have been studied as possible plant growth promoter under both natural and stressful conditions [8,56]. In the current study, an attempt has been made to isolate potent fungal endophytes from the spines and leaf segments of *S. xanthocarpum*. Of the 12 isolates, *M. caribbica* was studied in detail because of its ability to tolerate NaCl stress and efficiently promoted the growth of Waito-c rice. The growth of the Waito-c rice might be promoted through exogenous supply of vital plant growth hormones and secondary metabolites by *M. caribbica*. Also, *M. caribbica* has exhibited siderophore activity and efficiently solubilized the insoluble phosphate under laboratory conditions (Table 2). The ability of *M. caribbica* to exhibit siderophore activity and phosphate solubilization indicated that this species has the ability to improve iron and phosphate uptake by the host plant [11]. The results are in line with that of Sharma et al. [57] and Hamayun et al. [8], who have observed similar activities in their isolated plant growth promoting endophytes. The *M. caribbica* also secreted IAA, phenolics and falvonoids, which can promote plant immunity against abiotic stresses, including salinity [9]. DPPH radicals scavenging assay are commonly used assay to study the scavenging potential of the potent endophytes, our results showed that *M. caribbica* exhibited best DPPH radicals scavenging activity. Therefore, it is possible that *M. caribbica* has produced reductone, which might have reacted with the free radicals to alleviate salt stress and block radical chain reactions [58].

The endophyte produced a number of flavonoids, including *p*-hydroxybenzoic acid, genestin, diadzein, myricetin and caffeoyl-d-glucose. The flavonoids as an antioxidant are indeed important to promote stress tolerance in plants. In addition, genestien, *p*-hydroxybenzoic acid, diadzein, myrecitin and caffeoyl-d-glucose might have contributed toward the DPPH scavenging potential [59] of *M. caribbica* and thus helped the maize plants to withstand salt stress. Furthermore, the presence of flavonoids/phenols enables the endophyte *M. caribbica* to scavenge ROS that might be generated during exposure to NaCl stress [16]. The results suggested that under stress conditions, *M. caribbica* modulated the host plant system in order to detoxify the hazardous ROS rather enabling the host plant to avoid uptake of salts.

Maize plant is generally believed to be sensitive to salt stress [2], that is why exposure to salt stress in the present study has inhibited the growth of the maize seedlings. Likewise, the growth parameters (chlorophyll and carotenoid contents, reduced shoot and root growth, low plant weight on the fresh and dry bases, low RWCs and higher electrolytes leakage) in maize seedlings have significantly reduced, when exposed to salt stress. There are many reasons for severe plant growth reduction under saline conditions, but one of the important reasons might be declination in photosynthesis. Under salinity stress chloroplast accumulates excessive Na^+^ that inhibits PS-II activity, thus deters photosynthesis and ultimately growth rate [60]. Also, the decrease in plant RWC reflects on physiological drought that has been forcibly imposed on plants, as soil solution in saline areas is hypertonic in relation to cell sap [61]. In the current research project the inoculated *M. caribbica* helped the maize seedlings to stand against salt stress that was quite evident from the accumulation of pigments (chlorophyll and carotenoids content), decrease in EL, increased seedling growth and improvement in leaf RWC. The response of maize seedlings to selected salts (KCl, NaCl and K_2_SO_4_) in the current study was same.

Proline is an osmolyte that decreases the osmotic potential of the cell and the uptake of toxic ions [62]. Therefore, proline shows a major role in defending plants from osmotic stress [63]. In the present study, proline contents were greatly increased in maize plant inoculated with fungal endophyte *M. caribbica* under the saline environment. The results of increasing proline contents in maize plants inoculated with *M. caribbica* under saline conditions are in agreement with the result of Bagheri et al. [64]. Moreover, the improved level of proline content in plants under saline environment is due to the stimulation of proline biosynthesis, which improves protein turnover [63]. Considerable accumulation of proline in *Paecilomyces formosus* inoculated plants under salt stress also suggested a degeneration of ionic influx interior to the cells and hence rescued plants to keep its osmotic balance [65].

Another phenomenon that was affected by salt stress, include changes in endogenous phytohormones, i.e. high ABA and low IAA content. In the present study, high contents of IAA have been noticed in plants that have been inoculated with *M. caribbica* and subjected to salinity stress. Our results suggested that ABA and IAA have contrasting roles in plant response to salt stress. Maize seedlings associated with *M. caribbica* also tried to withstand the negative effects of salts by increasing the production of POD and catalase. Besides, the up-regulation of genes associated with CAT, POD, *M. caribbica* might also contribute toward the maize seedlings resistance against salinity stress by restoring the non-enzymatic antioxidant system [8,66,67].

## Availability of Data and Material

All the data are included in the manuscript.

## Supplementary Material

Supplementary Figure S1 and Table S1

## Abbreviations

ABA: abscisic acid
aPDA: potato dextrose agar
CF: culture filtrate
cfu: colony forming unit
ddH_2_O: double distilled water
DPPH: 2,2 diphenyl-1-picrylhydrazyl
EL: electrolyte leakage
GC/MS-SIM: gas chromatography-mass spectroscopy
IAA: indole acetic acid
ITS: internal transcribed spacer
OD: optical density
POD: peroxidase activity
PS-II: photosystem II
PVP: polyvinylpyrrolidone
ROS: reactive oxygen species
RWC: relative water content
SIM: selected ion monitoring
TE buffer: Tris buffer
