# Supplementary material for: A promising growth promoting Meyerozyma caribbica from Solanum xanthocarpum alleviated stress in maize plants
Source: Biosci Rep. 2019 Oct 25;39(10):BSR20190290. doi: 10.1042/BSR20190290 (PMC6822527; doi:10.1042/BSR20190290)
Supplement: Supplementary Figure S1 and Table S1 [file BSR-2019-0290_supp.pdf]

A promising growth promoting *Meyerozyma caribbica* from *Solanum xanthocarpum* alleviated stress in maize plants

Supplementary data

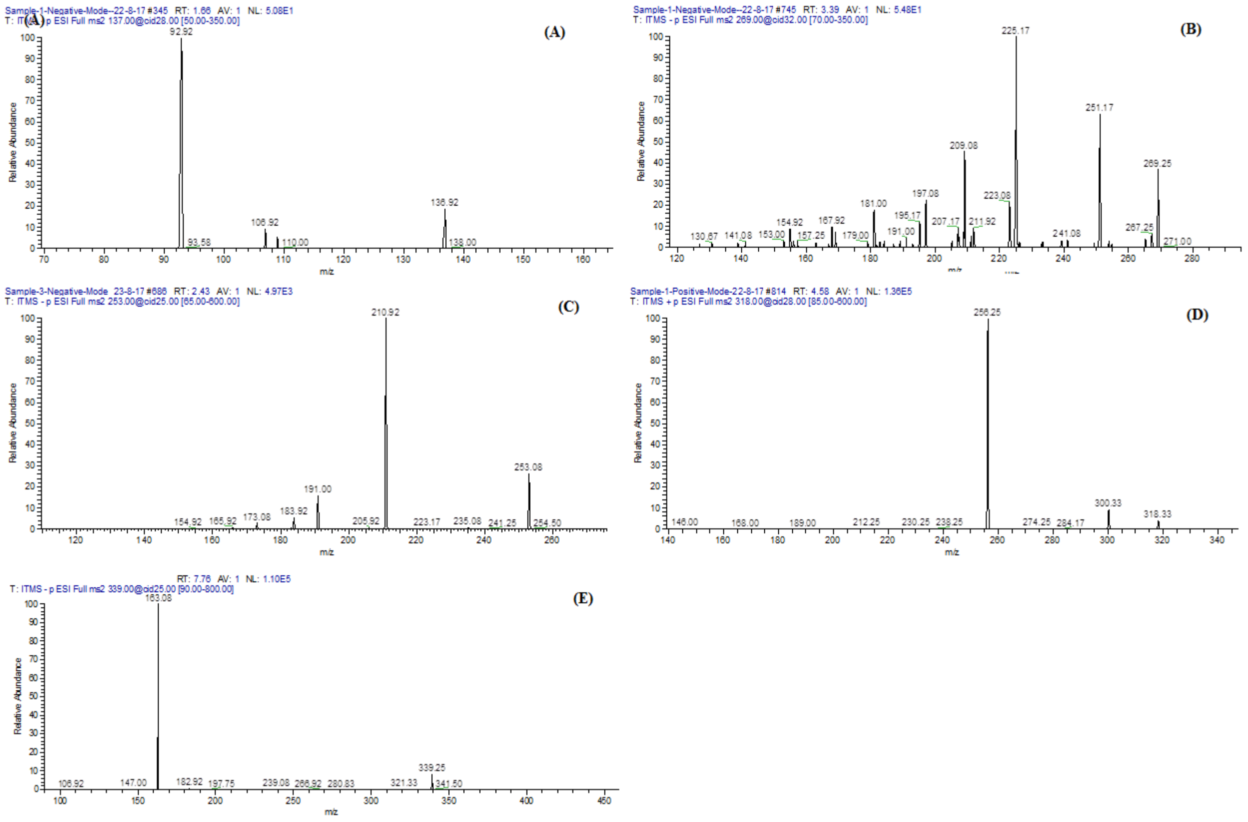

Figure S1: LC MS/MS chromatograms of SXSp1 culture filtrate.

Figure 4A represents LCMS/MS of p-hydroxbenzoic acid; Figure 4B represents LCMS/MS of genistien; Figure 4C represents LCMS/MS of diadzein; Figure 4D represents LCMS/MS of quercetagetin; Figure 4B represents LCMS/MS of caffeoyl-D-glucose.

Correlations

		Chla	Chlb	Carotenoid	EL	RWC	PFW	PDW	SL	RL	IAA	ABA	Proline	POD	CAT	Phenol	Flavonoids
Chla	Pearson Correlation	1	.975**	.711**	-.654**	.787**	.819**	.882**	.921**	.414*	.704**	-.799**	-.475*	-.715**	-.738**	.801**	.632**
	Sig. (2-tailed)		.000	.000	.001	.000	.000	.000	.000	.044	.000	.000	.019	.000	.000	.000	.001
	N	24	24	24	24	24	24	24	24	24	24	24	24	24	24	24	24
Chlb	Pearson Correlation	.975**	1	.659**	-.587**	.761**	.815**	.879**	.954**	.364	.659**	-.763**	-.517**	-.748**	-.758**	.740**	.559**
	Sig. (2-tailed)	.000		.000	.003	.000	.000	.000	.000	.080	.000	.000	.010	.000	.000	.000	.005
	N	24	24	24	24	24	24	24	24	24	24	24	24	24	24	24	24
Carotenoid	Pearson Correlation	.711**	.659**	1	-.802**	.848**	.804**	.876**	.742**	.791**	.938**	-.492*	.187	-.137	-.180	.916**	.915**
	Sig. (2-tailed)	.000	.000		.000	.000	.000	.000	.000	.000	.000	.015	.382	.524	.401	.000	.000
	N	24	24	24	24	24	24	24	24	24	24	24	24	24	24	24	24
EL	Pearson Correlation	-.654**	-.587**	-.802**	1	-.643**	-.544**	-.802**	-.538**	-.728**	-.892**	.759**	-.119	.302	.338	-.897**	-.893**
	Sig. (2-tailed)	.001	.003	.000		.001	.006	.000	.007	.000	.000	.000	.578	.152	.106	.000	.000
	N	24	24	24	24	24	24	24	24	24	24	24	24	24	24	24	24
RWC	Pearson Correlation	.787**	.761**	.848**	-.643**	1	.809**	.881**	.875**	.815**	.884**	-.452*	.107	-.243	-.237	.906**	.854**
	Sig. (2-tailed)	.000	.000	.000	.001		.000	.000	.000	.000	.000	.026	.619	.253	.265	.000	.000
	N	24	24	24	24	24	24	24	24	24	24	24	24	24	24	24	24
PFW	Pearson Correlation	.819**	.815**	.804**	-.544**	.809**	1	.850**	.881**	.475*	.734**	-.490*	-.142	-.257	-.308	.725**	.656**
	Sig. (2-tailed)	.000	.000	.000	.006	.000		.000	.000	.019	.000	.015	.509	.225	.143	.000	.001
	N	24	24	24	24	24	24	24	24	24	24	24	24	24	24	24	24
PDW	Pearson Correlation	.882**	.879**	.876**	-.802**	.881**	.850**	1	.914**	.710**	.918**	-.734**	-.103	-.452*	-.459*	.913**	.853**
	Sig. (2-tailed)	.000	.000	.000	.000	.000	.000		.000	.000	.000	.000	.632	.026	.024	.000	.000
	N	24	24	24	24	24	24	24	24	24	24	24	24	24	24	24	24
SL	Pearson Correlation	.921**	.954**	.742**	-.538**	.875**	.881**	.914**	1	.507*	.736**	-.598**	-.319	-.560**	-.564**	.775**	.637**
	Sig. (2-tailed)	.000	.000	.000	.007	.000	.000	.000		.011	.000	.002	.128	.004	.004	.000	.001
	N	24	24	24	24	24	24	24	24	24	24	24	24	24	24	24	24
RL	Pearson Correlation	.414*	.364	.791**	-.728**	.815**	.475*	.710**	.507*	1	.898**	-.318	.563**	.108	.142	.841**	.933**
	Sig. (2-tailed)	.044	.080	.000	.000	.000	.019	.000	.011		.000	.130	.004	.616	.509	.000	.000
	N	24	24	24	24	24	24	24	24	24	24	24	24	24	24	24	24
IAA	Pearson Correlation	.704**	.659**	.938**	-.892**	.884**	.734**	.918**	.736**	.898**	1	-.581**	.252	-.165	-.185	.971**	.985**
	Sig. (2-tailed)	.000	.000	.000	.000	.000	.000	.000	.000	.000		.003	.234	.441	.388	.000	.000
	N	24	24	24	24	24	24	24	24	24	24	24	24	24	24	24	24
ABA	Pearson Correlation	-.799**	-.763**	-.492*	.759**	-.452*	-.490*	-.734**	-.598**	-.318	-.581**	1	.465*	.752**	.739**	-.649**	-.546**
	Sig. (2-tailed)	.000	.000	.015	.000	.026	.015	.000	.002	.130	.003		.022	.000	.000	.001	.006
	N	24	24	24	24	24	24	24	24	24	24	24	24	24	24	24	24
Proline	Pearson Correlation	-.475*	-.517**	.187	-.119	.107	-.142	-.103	-.319	.563**	.252	.465*	1	.850**	.864**	.107	.348
	Sig. (2-tailed)	.019	.010	.382	.578	.619	.509	.632	.128	.004	.234	.022		.000	.000	.619	.096
	N	24	24	24	24	24	24	24	24	24	24	24	24	24	24	24	24
POD	Pearson Correlation	-.715**	-.748**	-.137	.302	-.243	-.257	-.452*	-.560**	.108	-.165	.752**	.850**	1	.980**	-.318	-.080
	Sig. (2-tailed)	.000	.000	.524	.152	.253	.225	.026	.004	.616	.441	.000	.000		.000	.130	.711
	N	24	24	24	24	24	24	24	24	24	24	24	24	24	24	24	24
CAT	Pearson Correlation	-.738**	-.758**	-.180	.338	-.237	-.308	-.459*	-.564**	.142	-.185	.739**	.864**	.980**	1	-.343	-.093

	Sig. (2-tailed)	.000	.000	.401	.106	.265	.143	.024	.004	.509	.388	.000	.000	.000		.101	.665
	N	24	24	24	24	24	24	24	24	24	24	24	24	24	24	24	24
Phenol	Pearson Correlation	.801**	.740**	.916**	-.897**	.906**	.725**	.913**	.775**	.841**	.971**	-.649**	.107	-.318	-.343	1	.957**
	Sig. (2-tailed)	.000	.000	.000	.000	.000	.000	.000	.000	.000	.000	.001	.619	.130	.101		.000
	N	24	24	24	24	24	24	24	24	24	24	24	24	24	24	24	24
Flavonoids	Pearson Correlation	.632**	.559**	.915**	-.893**	.854**	.656**	.853**	.637**	.933**	.985**	-.546**	.348	-.080	-.093	.957**	1
	Sig. (2-tailed)	.001	.005	.000	.000	.000	.001	.000	.001	.000	.000	.006	.096	.711	.665	.000	
	N	24	24	24	24	24	24	24	24	24	24	24	24	24	24	24	24

**. Correlation is significant at the 0.01 level (2-tailed).

*. Correlation is significant at the 0.05 level (2-tailed).
